# Cost-effectiveness of long-acting progestogens versus the combined oral contraceptives pill for preventing recurrence of endometriosis-related pain following surgery: an economic evaluation alongside the PRE-EMPT trial

**DOI:** 10.1136/bmjopen-2024-088072

**Published:** 2024-12-09

**Authors:** Melyda Melyda, Mark Monahan, Kevin G Cooper, Siladitya Bhattacharya, Jane P Daniels, Versha Cheed, Lee Middleton, Tracy E Roberts

**Affiliations:** 1 Health Economics Unit, Department of Applied Health Science, College of Medicine and Health, University of Birmingham, Birmingham, B15 2TT, UK; 2 NHS Grampian, Aberdeen Royal Infirmary, Foresterhill, Aberdeen, AB25 2ZN, UK; 3 School of Medicine Medical Sciences & Nutrition, University of Aberdeen, Foresterhill, Aberdeen, AB24 3FX, UK; 4 Nottingham Clinical Trials Unit, University of Nottingham, Nottingham, NG7 2RD, UK; 5 Birmingham Clinical Trials Unit, College of Medicine and Health, University of Birmingham, Edgbaston, Birmingham, B15 2TT, UK

**Keywords:** HEALTH ECONOMICS, GYNAECOLOGY, Healthcare Costs

## Abstract

**Objectives:**

To evaluate the cost-effectiveness of long-acting progestogens (LAP), including levonorgestrel-releasing intrauterine system (LNG-IUS) and depot-medroxyprogesterone acetate (DMPA), compared with the combined oral contraceptives pill (COCP) in preventing recurrence of endometriosis-related pain postsurgery.

**Design:**

Within-trial economic evaluation alongside a multicentre, pragmatic, parallel-group, open-label, randomised controlled trial (Preventing Recurrence of Endometriosis by means of Long-Acting Progestogen Therapy trial).

**Setting:**

Thirty-four UK hospitals recruiting participants from November 2015 to March 2019.

**Patients:**

Four hundred and five women aged 16–45 years undergoing conservative endometriosis surgery.

**Interventions:**

The ratio of 1:1 randomisation to receive LAPs (LNG-IUS or DMPA) or COCP.

**Main outcome measures:**

The primary evaluation was a cost-utility analysis based on cost per quality-adjusted life-year (QALY) gained at 3 years. We adopted a UK National Health Service perspective. Secondary analyses in the form of cost-effectiveness analysis based on a range of outcomes were also undertaken.

**Results:**

For the primary analysis, the COCP group incurred an additional cost of £533 (95% CI £52 to £983) per woman compared with LAPs. Treatment with COCP generated additional QALYs of 0.031 (95% CI −0.079 to 0.139) compared with the LAP group over 36-month follow-up. The incremental cost-effectiveness ratio for COCP compared with LAPs is therefore approximately £17 193 per QALY. The probabilistic sensitivity analysis suggested that there was a 54.7% probability that COCP would be cost-effective at the £20 000/QALY threshold. The secondary analyses revealed results more in favour of LAPs.

**Conclusion:**

Although the COCP has a slightly higher probability of being cost-effective at £20 000/QALY threshold, there remains considerable uncertainty, with only marginal differences in outcomes between the two treatments. The lower rates of further surgery and second-line medical treatment for women allocated to LAPs may make this option preferable for some women.

**Trial registration number:**

ISRCTN 97865475.https://www.isrctn.com/ISRCTN97865475

**STRENGTHS AND LIMITATIONS OF THIS STUDY**

- The study used prospective data collection within a pragmatic randomised controlled trial, offering valuable real-world insights into patient preferences and treatment responses.
- The primary economic outcome measure, EuroQol-5 Dimensions, a five-level version questionnaire, had a high completeness rate of 83% at 36 months.
- Multiple sensitivity analyses were conducted to explore the impact of different assumptions and inputs.
- While the trial-based economic evaluation provides cost-effectiveness insights, treatment crossover, women’s preferences, risk of further surgery and use of second-line medical treatments may have influenced our result and should be considered before making definitive recommendations.

## Introduction

 Endometriosis is a chronic condition affecting over 190 million women of reproductive age globally. It is characterised by abnormal tissue growth resembling uterine lining which occurs outside the uterus.^1 2^ This condition imposes a substantial socioeconomic burden, with the UK economy incurring an annual cost of £8.2 billion, primarily attributed to productivity losses (65%) and healthcare expenses.^3^ The loss of productivity is closely linked to endometriosis-related symptoms, particularly significant pelvic pain.^4^ There is also an associated 19% reduction in overall quality of life for women experiencing endometriosis-associated symptoms compared with those in optimal health.^3^

Surgical treatment is the predominant treatment for alleviating endometriosis-related pain and symptoms but it is associated with a high recurrence rate leading to repeat surgery for 27% of patients within 5 years.^5^ Clinical practice guidelines recommend the use of hormonal therapy to manage endometriosis-related pain.^6 7^ This includes the combined oral contraceptives pill (COCP), which requires daily oral intake, and long-acting progestogens (LAPs), such as depot medroxyprogesterone acetate (DMPA), which is administered every 3 months, and the levonorgestrel-releasing intrauterine system (LNG-IUS).^6 7^ The LNG-IUS is licensed for up to 8 years for contraception and up to 5 years for heavy menstrual bleeding.^8^ The comparative effectiveness and cost-effectiveness of these treatment regimens in preventing postsurgical endometriosis-related pain and their effectiveness in reducing repeat surgery recurrence remain unclear. Previous research has explored the cost-effectiveness of various endometriosis treatments,^9 10^ but none has specifically evaluated the role of LAP and COCP to reduce pain and symptoms postsurgery.

The Preventing Recurrence of Endometriosis by means of Long-Acting Progestogen Therapy (PRE-EMPT) trial aims to fill this gap with the primary outcome of the clinical trial being a pain as measured by the pain domain of the Endometriosis Health Profile 30 (EHP-30) questionnaire at 3 years post-randomisation. The findings from the clinical trial have been published in the *British Medical Journal*.^11^ This current paper presents the economic evaluation conducted alongside the trial. The primary aim of this paper is to assess the cost-effectiveness of LAPs or COCP in preventing the recurrence of pain in women undergoing conservative surgery for endometriosis, in terms of additional cost per quality-adjusted life-year (QALY) gain. The secondary objectives were to assess the cost-effectiveness for a range of outcomes in natural units, including cost per year of full capability, cost per pain score reduction and cost per case of treatment failure avoided for both treatment options.

## Methods

PRE-EMPT was a multicentre, pragmatic, parallel-group, open-label, randomised controlled trial (RCT). Details of the trial, including the economic evaluation plan and protocol, have been published elsewhere.^11^,^13^ Briefly, 405 women aged 16–45 years with symptoms suggestive of endometriosis and scheduled for a diagnostic laparoscopy with concurrent or previous conservative surgery were recruited and randomised across 34 hospitals in the UK from November 2015 to March 2019, of which 205 receiving LAPs (91 to LNG-IUS and 114 to DMPA) and 200 receiving COCP. Exclusion criteria included infertility, immediate plans to conceive, elective surgery for deep disease or endometrioma, contraindications to hormonal treatment and suspicion of malignancy.^11 13^

The primary economic analysis was based on the outcome of the QALY gained over 36 months and the secondary analysis was based on the outcomes of years of full capability, the EHP-30 pain score reduction and treatment failure avoided. All statistical analyses were performed using Stata V.17 (StataCorp LP, College Station, TX, USA).

### Measurement and valuation of outcomes

All the outcome measures were collected at baseline, 6, 12, 24 and 36 months after randomisation. For the primary economic evaluation, the QALY scores were generated through the EuroQol-5 Dimensions, a five-level version (EQ-5D-5L) questionnaire. The responses were converted to index scores using a crosswalk value set (EEPRU dataset) for the UK population to map from the EQ-5D-5L to EQ-5D-3L valuation set.^14^ QALYs were then estimated for each participant using the approach of area-under-the-utility curve assuming linear interpolation between the five utility measurements,^15^ where the utility score associated with a certain health state was multiplied by the duration of time spent in that health state. Adjustments were made for any differences between the groups in their initial EQ-5D-5L scores using a multiple linear regression method to minimise the potential bias from an imbalance in the baseline.^16^ The health utility values and QALYs obtained during the 36-month follow-up were analysed by trial allocation group and time point.

In the secondary analysis based on capability, the years of full capability were generated from the ICEpop CAPability measure for Adults (ICECAP-A) questionnaire. This measure combines scores with time to represent the total capability available over time, employing an approach akin to the area-under-the-curve method used for calculating QALYs. The ICECAP-A is a validated capability measure for the adult population, focusing on well-being in a broader sense.^17^ To mitigate potential bias, adjustments for any initial differences between groups in their ICECAP-A scores were made using the multiple linear regression method.^18^

The secondary analysis also considered the differences in pain scores, which were determined by the changes in the EHP30 pain domain score from the baseline to 3-year follow-up. The EHP-30 questionnaire is a patient-reported outcome measure to assess health-related quality of life in endometriosis.^19^ The core components of this instrument encompass pain, control and powerlessness, social support, emotional well-being and self-image scale scores. Only the pain-domain score was considered for the secondary analysis outcome. The pain domain consists of 11 questions, with overall 0 as the best outcome to 100 pain score as the worst score.^20^ The pain score changing was the difference between the pain score at the baseline and at the 36-month follow-up time.

The final outcome in secondary analysis centred on treatment failure avoided. Treatment failure was classified as patients who had further surgery for endometriosis, hysterectomy and laparoscopy or who used second-line treatment of gonadotropin-releasing hormone analogue (GnRHa) for symptom management.

### Resource use and costs

Healthcare resource utilisation data were gathered alongside the trial at various intervals: baseline, 6, 12, 24 and 36 months post-randomisation, employing the PRE-EMPT follow-up questionnaires. Information on the direct cost to the healthcare provider was obtained for medications, including the type of hormonal treatment and painkiller used, hospital and primary consultations, investigation procedures (laparoscopy, hysteroscopy and ultrasound scan) and further surgical procedures (surgery for endometriosis and hysterectomy), and collected prospectively in the study. The questionnaires also captured indirect nonmedical costs, such as income or productivity losses attributable to endometriosis. This aspect was assessed using the human capital approach, wherein the time lost due to endometriosis symptoms (measured in days) was multiplied by average gross wage estimates.^21^ This method was deemed appropriate since the majority of work absences tended to be of relatively shorter duration.

Some pragmatic assumptions were required in measuring healthcare resource use and costs within the trial, including:

- All healthcare visits, such as those to a general practitioner or hospital, were included, although some may not have been related to endometriosis symptoms.
- If a participant reported undergoing ‘surgery for endometriosis’ or ‘hysterectomy’ and specified ‘laparoscopy’ simultaneously, it was considered a single procedure (eg, surgery for endometriosis by laparoscopy or laparoscopic hysterectomy).
- In the event of treatment switching or if not reported, it was assumed to occur midway between follow-up points for costing purposes.

Table 1 presents the relevant items of resources used, their associated unit costs and the source from which these costs were obtained. All costs were reported in 2021–2022 British pounds. Costs were inflated where necessary, using the Hospital and Community Health Services Pay and Prices Index.^22^

**Table 1:** Unit cost of resource use items (2021–2022 prices)

Resource use items	Unit cost (£)	HRG code/details	Source
Medication			
COCP (Microgynon) Ethinylestradiol 30 µg, Levonorgestrel 150 µg	0.94	Per pack (for 28 days preparation)	BNF 84^32^
Levonogestrel (Mirena) 20 µg/24 hours intrauterine device	88	Per device	BNF 84^32^
Medroxyprogesterone acetate (Depo-Provera) 150 mg/1 mL suspension for injection vials	6.01	Per vial	BNF 84^32^
Triptorelin (Decapeptyl SR) 3 mg (GnRHa)	69	Per vial	BNF 84^32^
Pain relief medication	0.97	Weighted average of participant pain relief medication	BNF 84^32^
Primary care visit			
GP consultation (10 min)	45.17		PSSRU 2022^22^
Further surgery			
Removal of polyps	4369.71	MA09B	NHS Reference cost 2020/21^33^
Removal of fibroids	4369.71	MA09B	NHS Reference cost 2020/21^33^
Endometrial ablation	1416.51	MA12Z	NHS Reference cost 2020/21^33^
Laparoscopic hysterectomy	5935.16	MA08B	NHS Reference cost 2020/21^33^
Surgery for endometriosis via laparoscopy	4369.71	MA09B	NHS Reference cost 2020/21^33^
Test/investigations			
Laparoscopy	3280.88	MA10Z	NHS Reference cost 2020/21^33^
Ultrasound	71.90	RD40Z	NHS Reference cost 2020/21^33^
Hysteroscopy	521.82	MA31Z	NHS Reference cost 2020/21^33^
Follow-up after surgery	235.39	WF01A	NHS Reference cost 2020/21^33^
Productivity loss			
Full-time employee work absence	640*	Per week†	Office for National Statistics 2022^21^
Part-time employee work absence	228*	Per week†	Office for National Statistics 2022^21^

* **Excluding the employer National Insurance NI and pension contribution due to limited data availability.

† Included in sensitivity analysis only.

BNF, British National Formulary; COCP, combined oral contraceptives pill; GnRHagonadotropin-releasing hormone analogue GPgeneral practitionerHRG, Healthcare Resource GroupNHSNational Health ServicePSSRU, Personal Social Services Research Unit

### Missing data

Multiple imputation techniques were used to handle missing costs and missing EQ-5D-5L, ICECAP-A and EHP-30 pain domain data at each follow-up time point.^23 24^ Costs were imputed at the total cost level for each cost category. Outcome and resource use data, and therefore costs, were considered missing if participants did not complete and return their follow-up questionnaire.

Multiple imputation was performed by the predictive mean matching method to the closest neighbour based on the treatment group with a chained equation to account for the non-normality of the distribution of costs and the outcome values for missing total costs and missing outcomes.^25^ The imputation model was based on the treatment group and used 20 imputed datasets. Subsequently, Rubin’s rules were applied to combine the results obtained from the multiple imputed datasets.^24^ The imputed data were used to inform the base-case and sensitivity analyses unless specified otherwise.

### Economic evaluation analysis

The primary base-case economic analysis adopted a cost-utility analysis framework, conducted from the perspective of the UK National Health Service (UK NHS) and personal social services. This analysis aimed to evaluate the gains in QALYs relative to the costs of different interventions.

The secondary economic evaluation was the cost-effectiveness analysis, where the health consequences were measured in a natural unit as YFCs, EHP30 pain score change and treatment failure avoided. Incremental cost-utility and cost-effectiveness analyses were conducted to calculate the incremental cost-effectiveness ratio (ICER), representing the cost per outcome between the two trial groups. Both costs and QALYs were discounted at a rate of 3.5% per annum, following recommendations from the National Institute for Health and Care Excellence (NICE).^26^ As the cost and QALY data exhibited skewness, all estimates were presented as means with bootstrapped 95% CIs, each generated through 5000 replications.

### Deterministic sensitivity analysis

A series of deterministic sensitivity analyses were carried out to assess the robustness of the base-case results:

1. Undiscounted analysis: this analysis presented the undiscounted costs and outcomes.
2. Partial societal perspective analysis: this analysis assessed the impact of including work-related costs of patients.
3. Additional analysis: this analysis incorporated costs of other types of surgery mentioned by participants (removal of fibroids, removal of polyps and endometrial ablation).
4. Subgroup analysis: this analysis evaluated the cost-effectiveness of the COCP with each of the LAP’s subgroups: COCP versus LNG-IUS and COCP versus DMPA.
5. Complete-case analysis: the analysis was re-run using only participants with complete cost and outcome data.

### Probabilistic sensitivity analysis

A probabilistic sensitivity analysis (PSA) was undertaken for the base-case analysis, by jointly bootstrapping mean cost and outcome differences to generate 5000 paired ICER estimates. The results were plotted in a cost-effectiveness plane that comprises four quadrants: north-east (NE), north-west (NW), south-east (SE) and south-west (SW), each representing a different cost-effectiveness scenario.^27^ The NE quadrant represented situations where the intervention is both more effective and more costly than the comparator. The SE quadrant indicates that the intervention is both more effective and cheaper than the comparator, implying that the intervention is dominant and hence the preferred option. The SW quadrant indicates the intervention is less effective and less costly while the NW quadrant suggests that the intervention is less effective and more costly than the comparator.^27 28^

Results were also estimated by constructing cost-effectiveness acceptability curves (CEACs) to reflect the uncertainties in the cost-effectiveness value where appropriate.^29^ For the primary economic analysis, the CEAC shows the probability of COCP and/or LAPs being cost-effective at different cost-per-QALY thresholds. In the UK, interventions are typically considered cost-effective if the cost per QALY gained is equal to or less than £20 000.^26^

## Results

Out of a total of 405 participants included in the trial, 200 were randomised to COCP and 205 women were randomised to LAP. Of those randomised to LAP, 91 were either allocated based on their preference or were randomised to LNG-IUS and 114 to DMPA. The follow-up rate at 36 months was 83% across all groups.^11 13^The response rate for the participant-completed outcome using the EQ-5D-5L questionnaire and the utility score at each follow-up time point is presented in online supplemental tables S1 and S2. Resource use, disaggregated costs and mean total costs are presented in online supplemental tables S3–S5.

At the 36-month time point at the end of the study, complete data were available for approximately 85% of women in both groups. Complete economic data from baseline to 36 months from EQ-5D-5L questionnaires were available for 214 (52.84%) participants.

At baseline, participants in the LAPs group had a slightly lower average starting EQ-5D-5L score than those in the COCP group (0.640 and 0.643, respectively). However, by 36 months, participants in the LAPs group showed a slightly higher score compared with those in the COCP group (0.697 and 0.687, respectively). The mean-adjusted imputed QALY difference between the two groups was 0.031 (95% CI −0.079 to 0.139), favouring the COCP group.

Women in the LAPs group underwent fewer surgical procedures, on average, resulting in fewer follow-up episodes compared with the COCP group. They also had fewer instances of using analgesics, second-line medical treatment (GnRHa) and ultrasound scans. However, they incurred more primary care visits (likely due to the need for injections every 3 months with DMPA) and an increased number of diagnostic procedures compared to the COCP group. The COCP group underwent more surgery for recurrent endometriosis and hysterectomy procedures compared with the LAPs group, resulting in the additional cost of approximately £505 per woman. Further follow-up visits after these surgeries incurred an extra cost of £160 compared with the LAPs group.

Consequently, in the base-case analysis (table 2), the average costs were £2470 per woman in the COCP group compared with £1937 per woman in the LAP group. Thus, the COCP group was estimated to be £533 (95% CI 52 to 983) per woman more costly but offered slightly higher QALYs by 0.031 (95% CI −0.079 to 0.139) compared with the LAPs group over the 36-month follow-up. This resulted in an ICER of £17 193 per QALY.

**Table 2:** Result of base-case analysis and deterministic sensitivity analyses

	Mean cost	Mean effect (QALY)	Bootstrap difference, mean incremental cost (95% CI)	Bootstrap difference, mean incremental effect (95% CI)	ICER
Base-case analysis					
LAPs	£1937	1.936	£533 (£52 to £984)	0.031 (−0.079 to 0.139)	£17 193
COCP	£2470	1.968			
Deterministic sensitivity analysis					
1. Undiscounted cost and outcome					
LAPs	£2012	2.005	£466 (£−51 to £949)	0.032 (−0.086 to 0.146)	£14 562
COCP	£2477	2.037			
2. Partial societal perspective					
LAPs	£4546	1.936	£773 (£229 to £1297)	0.031 (−0.079 to 0.139)	£24 935
COCP	£5319	1.968			
3. Including the cost of other types of surgery mentioned by participants (removal of fibroids, removal of polyps and endometrial ablation)					
LAPs	£2006	1.936	£631 (£117 to £1128)	0.031 (−0.079 to 0.139)	£20 354
COCP	£2637	1.968			
4a. Subgroup analysis: LNG-IUS vs COCP					
LNG-IUS	£2336	1.953	£135 (£−709 to £769)	0.015 (−0.090 to 0.176)	£9009
COCP	£2471	1.968			
4b. Subgroup analysis: DMPA vs COCP					
DMPA	£1619	1.924	£851 (£391 to £1,269)	0.044 (−0.091 to 0.180)	£19 232
COCP	£2471	1.968			

COCPcombined oral contraceptives pillDMPAdepot medroxyprogesterone acetateICERincremental cost-effectiveness ratioLAPslong-acting progestogensLNG-IUSlevonorgestrel-releasing intrauterine systemQALYquality-adjusted life-year

### Sensitivity analysis

**Figure 1:**
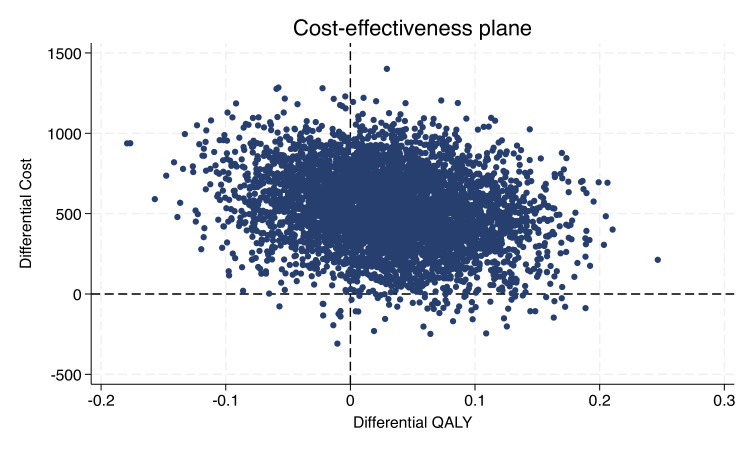
Cost-utility analysis from the UK NHS and personal social services perspective: cost-effectiveness plane showing 5000 bootstrapped replicates of the incremental cost-effectiveness ratio. QALY, quality-adjusted life-year.

**Figure 2:**
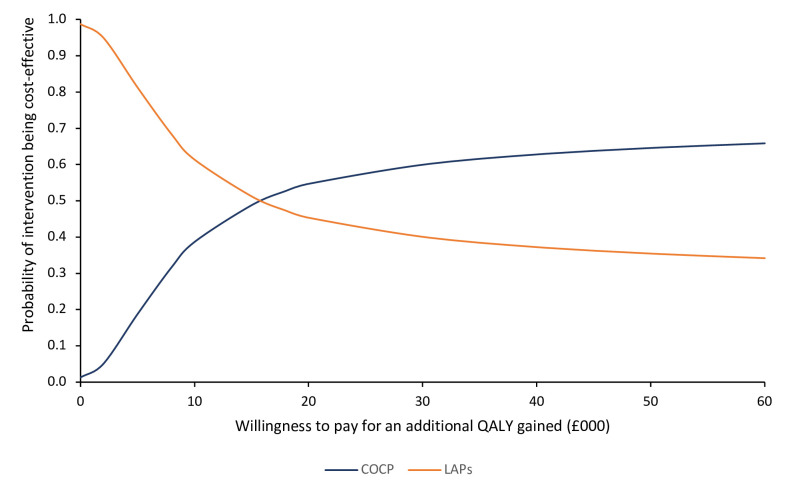
Cost-effectiveness acceptability curve for COCP compared with LAPs. COCP, combined oral contraceptives pill; LAPs, long acting progestogens; QALY, quality-adjusted life-year.

Table 2 presents the results of the deterministic sensitivity analysis. Among most analyses, COCP consistently yielded higher costs but greater QALYs compared with LAPs. These findings were also consistent with the subgroup analyses comparing COCP with LAP subgroups. However, in the complete case analysis (see online supplemental table S6), COCP was associated with higher costs and fewer QALYs, but this was limited by a smaller sample size and missing data, thereby affecting the robustness of this finding. The cost-effectiveness plane represents the PSA results (figure 1). The majority of the points lie in the NE quadrant from the origin, indicating that COCP was more costly and more effective than LAPs. The CEAC (figure 2) shows that the probability of the COCP intervention being considered cost-effective is approximately 54.7% at a threshold of £20 000 per QALY.

### Cost-effectiveness analysis

The results of a range of secondary analyses, which took the form of cost-effectiveness analyses based on single outcomes in natural units are presented in online supplemental tables S7 and S8. Complete economic data for ICECAP-A and EHP-30 pain were available for 147 (36.23%) and 212 (52.34%) participants, respectively.

Based on the specific single outcomes explored, these analyses show results different from those reported in the primary analysis.

- The mean-adjusted years of full capabilities difference for imputed data favoured the LAP group by 0.0034 (95% CI −0.0562 to 0.0525).
- The reduction in pain score between baseline and 36 months also favoured LAP, with a mean difference of 0.145 (–4.509 to 4.182).
- Fewer treatment failures were observed in the LAP group compared with the COCP group (16.59% vs 20.5%, respectively), resulting in a mean difference of 0.039 (95% CI −0.121 to 0.034).

Table 3 presents the summary of these cost-effectiveness results that suggest that LAPs are cheaper and more effective in achieving these individual outcomes than COCP. Thus, the COCP intervention was dominated by the LAPs intervention for these single outcomes.

**Table 3:** Cost per point change in secondary outcome results

Treatment groups	Mean cost	Mean effect	Bootstrap difference, mean incremental cost (95% CI)	Bootstrap difference, mean incremental effect (95% CI)	ICER
Years of full capability					
LAPs	£1937	2.326	£533 (£52 to £983)	−0.006 (−0.092 to 0.0762	Dominated
COCP	£2471	2.32			
EHP-30 pain domain score reduction					
LAPs	£1937	23.549	£533 (£52 to £983)	−0.145 (−4.509 to 4.182	Dominated
COCP	£2471	23.403			
Treatment failure avoided					
LAPs	£1937	0.166	£533 (£52 to £983)	0.039 (−0.035 to 0.113)	Dominated
COCP	£2471	0.205			

COCPcombined oral contraceptivesEHP-30Endometriosis Health Profile 30ICERincremental cost-effectiveness ratioLAPslong-acting progestogens

## Discussion

### Principal findings

The primary analysis based on outcomes in terms of QALYs showed that the cost for COCP is more than that for LAP, averaging £2470 per participant compared with £1937. The difference (£533, 95% CI £52 to £983) is primarily due to the cost of further surgeries for endometriosis or hysterectomy. COCP yields a slight increase in QALYs of 0.031 (95% CI −0.079 to 0.139) over 36 months. Thus, the ICER for treatment with COCP compared with LAP is £17 193 per QALY. This falls within the acceptable threshold of £20 000 per QALY recommended by NICE.^26^ Thus, the primary analysis suggests that COCP would be deemed cost-effective. However, the PSA indicated a 54.7% probability that COCP is more cost-effective than LAP in terms of cost per QALY gained. This probability is only slightly above 50%, highlighting a considerable level of uncertainty regarding which treatment is truly more cost-effective.

In the secondary analyses where the focus is a range of single outcomes reported in natural units, rather than an overall outcome of quality of life, there is a reversal of these results. LAPs demonstrated better outcomes compared with the COCP group across all secondary measures: years of full capability, EHP-30 pain domain score change and treatment failure avoided. This discrepancy between the primary and secondary outcomes suggests that while COCP may be cost-effective in terms of QALYs, the other clinical effectiveness of LAPs could offer additional benefits that are not captured by the QALY metric. As such, clinicians and decision-makers need to consider the results of the economic evaluation alongside the broader clinical context in choosing the most appropriate treatment for individual women.

### Strengths and limitations of the study

This is the first economic evaluation conducted alongside an RCT to assess the cost-effectiveness of any medical treatment postsurgery, including the COCP and LAPs in preventing the recurrence of endometriosis-related pain postsurgery. A strength of the study is the prospective data collection for both cost and outcome data. The primary outcome relies on QALYs measured using the EQ-5D-5L, for which there was a high completeness rate of 83% at 36 months. Extensive sensitivity analyses were performed to ensure the robustness of the findings and to explore the impact of different assumptions and inputs.

In terms of limitations, the 3-year follow-up duration may not fully capture the chronic nature of endometriosis, which can recur until menopause.^30^ Additionally, healthcare resource use information relied on self-reported data, which can be susceptible to under-reporting.^31^

The use of QALYs also may not encompass all outcomes crucial to women and their treatment pathways. Although additional analyses were carried out based on the secondary outcomes, the high rate of missing data for these outcomes hinders robust interpretation. Furthermore, the high rates of discontinuation and treatment switching among participants presented challenges in attributing outcomes to specific treatments. Nonetheless, employing an intention-to-treat analysis, conducting sensitivity analyses and collecting data at various time points served to mitigate these challenges. This pragmatic approach offers insights into real-world clinical scenarios, reflecting patient preferences and responses that impact treatment decisions.

This study’s main analysis also considered only the UK NHS and personal social services perspective, due to constraints in gathering comprehensive data required for a full societal analysis. However, a sensitivity analysis from a partial societal perspective was also conducted to explore the wider cost implications and provide additional context. Finally, a detailed LAPs subgroup analysis comparing LNG-IUS and DMPA to determine the more cost-effective option was not considered in this study, as it was beyond the scope of our primary research question and the design of the PRE-EMPT trial.^11 13^

### Comparison with previous research

This study is unique in its focus on the cost-effectiveness of COCP versus LAPs for preventing the recurrence of endometriosis-related pain in women who have undergone surgery. While existing studies have analysed the cost-effectiveness of various treatment approaches for endometriosis, none has specifically addressed the comparison between LAPs and COCP in this context via an RCT.^9 10^

It is also important to note that the main QALY result and ICER value in this study were slightly different from what we reported in another publication,^13^ although we did not find substantial differences in the overall results. The main findings remain consistent: COCP is more expensive but leads to higher QALYs gained compared with LAP. These differences are due to the use of the different crosswalk value set (EEPRU dataset)^14^ to map from EQ-5D-5L to EQ-5D-3L valuation set in this current analysis, as recommended by NICE.^26^

### Implications for policy

While the primary trial-based economic evaluation suggests a slightly higher probability of COCP being cost-effective compared with the LAPs in terms of cost per QALY gained, there remains considerable uncertainty regarding which treatment is more cost-effective. Additionally, the COCP group is also associated with an increased risk of further major surgery for recurrent endometriosis and hysterectomy which may influence decision-making by women and their healthcare practitioners. In terms of outcomes, both options offer similar benefits in terms of QALYs, with only marginal differences. Some women may prefer LAPs due to past experiences and the acceptability of their invasiveness balanced by the lower risk of further surgery. Therefore, thoroughly discussing both options with women is recommended.

### Recommendations for future research

Given the complexity of treating endometriosis and the various influencing factors such as patient adherence, providing a single recommendation with respect to the economic impact of COCP versus LAPs is challenging. Future research should focus on evaluating newer hormonal and non-hormonal methods of controlling endometriosis-related symptoms and the progression of the disease itself. Assessing the costs associated with these new treatments is essential, as well as identifying strategies to reduce rates of treatment attrition and improve compliance. Therefore, continued research is needed to identify opportunities for more effective and cost-efficient treatments.

## supplementary material

10.1136/bmjopen-2024-088072 online supplemental file 1

## Data Availability

Data are available upon reasonable request.
