# Supplementary material for: Cost-effectiveness of long-acting progestogens versus the combined oral contraceptives pill for preventing recurrence of endometriosis-related pain following surgery: an economic evaluation alongside the PRE-EMPT trial
Source: BMJ Open. 2024 Dec 9;14(12):e088072. doi: 10.1136/bmjopen-2024-088072 (PMC11628948; doi:10.1136/bmjopen-2024-088072)
Supplement: online supplemental file 1 [file bmjopen-14-12-s001.docx]

**Supplementary materials**

**Table S1. The primary outcome (EQ-5D-5L questionnaire) response rates**

| **Time point** | **LAPs group (N=205),**  **n (%)** | **COCP group (N=200),**  **n (%)** | **Total** | **Missing** |
| --- | --- | --- | --- | --- |
| **EQ-5D-5L questionnaire** | | | | |
| Baseline | 198 (96.58) | 190 (95) | 388 | 17 |
| Month 6 | 160 (78.05) | 149  74.5) | 309 | 96 |
| Month 12 | 151 (73.66) | 152 (76) | 303 | 102 |
| Month 24 | 157 (76.59) | 141 (70.5) | 298 | 107 |
| Month 36 | 176 (85.85) | 167 (83.5) | 343 | 62 |
| Complete case baseline to month 36 | 115 (56.09) | 99 (49.5) | 214 | 191 |

**Table S2. Utility and QALY estimates: EQ-5D-5L scores**

| **Time point** | **LAPs group (N=205)** | | **COCP group (N=200)** | | **Bootstrap adjusted mean difference (95% CI)** |
| --- | --- | --- | --- | --- | --- |
|  | **n** | **Mean (SD)** | **n** | **Mean (SD** |  |
| Baseline EQ-5D-5L score | 198 | 0.640 (0.227) | 190 | 0.643 (0.232) | 0.002  (-0.045 to 0.047) |
| Month 6 EQ-5D-5L score | 160 | 0.672 (0.241) | 149 | 0.680 (0.273) | 0.008  (-0.043 to 0.063) |
| Month 12 EQ-5D-5L score | 151 | 0.667 (0.274) | 152 | 0.668 (0.255) | 0.012  (-0.042 to 0.068) |
| Month 24 EQ-5D-5L score | 157 | 0.667 (0.282) | 141 | 0.687 (0.266) | 0.018  (-0.039 to 0.068) |
| Month 36 EQ-5D-5L score | 176 | 0.697 (0.250) | 167 | 0.687 (0.278) | -0.0114  (-0.071 to 0.041) |
| Total complete case QALYs | 115 | 1.965 (0.604) | 99 | 1.949 (0.652) | -0.016  (-1.537 to 0.095) |
| Total imputed QALYs | 205 | 1.936 (0.552) | 200 | 1.968 (0.582) | 0.031  (-0.079 to 0.139) |

**Table S3. Mean resource use across trial groups**

| **Resource item** | **LAPs group (N=205)** | | **COCP group (N=200)** | | **Bootstrap difference,**  **mean difference (95% CI)** |
| --- | --- | --- | --- | --- | --- |
|  | **Mean (SD)** | **n** | **Mean (SD)** | **n** |  |
| **Health care visit** | | | | | |
| GP visit | 7.28 (4.88) | 121 | 6.64 (6.36) | 110 | -0.64 (-1.97 to 0.96) |
| Gynaecology follow up after surgery visit | 0.62 (1.20) | 121 | 1.35 (2.42) | 110 | 0.73 (0.29 to 1.23) |
| **Medication** | | | | | |
| Combined oral contraceptives pill (COCP) | 3.92 (7.32) | 121 | 18.63 (12.07) | 110 | 14.71 (12.14 to 17.52) |
| Long-acting progestogens (LAPs) | 4.44 (4.21) | 121 | 0.96 (2.14) | 110 | -3.47 (-4.33 to -2.64) |
| Levonorgestrel releasing intrauterine system (LNGIUS) | 0.63 (0.57) | 121 | 0.21 (0.41) | 110 | -0.42 (-0.55 to -0.29) |
| Depot medroxyprogesterone acetate (DMPA) | 3.81 (4.52) | 121 | 0.76 (2.14) | 110 | -3.06 (-3.94 to -2.18) |
| Gonadotropin releasing hormone analogue (GnRHa) | 0.94 (2.57) | 121 | 0.98 (2.99) | 110 | 0.04 (-0.62 to 0.85) |
| Painkiller | 16.10 (13.67) | 121 | 23.07 (19.59) | 110 | 6.97 (2.81 to 11.82) |
| **Further procedures** | | | | | |
| Surgery for endometriosis | 0.16 (0.47) | 121 | 0.22 (0.42) | 110 | 0.06 (-0.06 to 0.17) |
| Hysterectomy combined | 0.07 (0.26) | 121 | 0.10 (0.30) | 110 | 0.03 (-0.04 to 0.10) |
| **Test/investigations** | | | | | |
| Laparoscopy | 0.07 (0.28) | 121 | 0.06 (0.23) | 110 | -0.01 (-0.08 to 0.04) |
| Ultrasound scan | 0.04 (0.20) | 121 | 0.07 (0.32) | 110 | 0.03 (-0.03 to 0.11) |
| Hysteroscopy | 0.02 (0.18) | 121 | 0 (0) | 110 | -0.02 (-0.06 to 0) |
| Productivity loss | | | | | |
| Days taken off-paid work | 13.38 (20.19) | 109 | 14.89 (25.56) | 96 | 1.52 (-4.81 to 7.85) |
| Days taken off-unpaid work | 11.19 (16.75) | 36 | 17.47 (30.56) | 36 | 6.28 (-4.48 to 18.23) |

**Table S4. Disaggregated costs by trial groups (£) (2021 – 22 prices)**

| **Resource item** | **LAPs group (N=205)** | | **COCP group (N=200)** | | **Bootstrap difference, mean cost difference (95% CI)** |
| --- | --- | --- | --- | --- | --- |
|  | **Mean (SD)** | **n** | **Mean (SD)** | **n** |  |
| **Health care visit** | | | | | |
| GP visit | 317.16 (168.13) | 205 | 290.88 (212.03) | 200 | -26.29 (-63.51 to 9.89) |
| Follow-up visit after surgery | 141.33 (213.19) | 205 | 300.48 (414.22) | 200 | 159.14 (98 to 223.91) |
| **Medication** | | | | | |
| Combined oral contraceptives pill (COCP) | 3.56 (5.16) | 205 | 17.06 (8.24) | 200 | 13.50 (12.09 to 14.77) |
| Long-acting progestogens (LAPs) | 76.71 (30.43) | 205 | 21.65 (27.05) | 200 | -55.05 (-60.72 to -49.20) |
| Levonorgestrel releasing intrauterine system (LNG-IUS) | 54.79 (38.23) | 205 | 17.39 (26.17) | 200 | -37.40 (-44.31 to -31.39) |
| Depot medroxyprogesterone acetate (DMPA) | 21.92 (20.47) | 205 | 4.26 (9.28) | 200 | -17.66 (-20.71 to -14.51) |
| Gonadotropin releasing hormone analogue (GnRHa) | 63.82 (135.54) | 205 | 64.98 (150.94) | 200 | 1.16 (-25.33 to 30.03) |
| Painkiller | 15.11 (10.01) | 205 | 21.79 (13.99) | 200 | 6.89 (4.31 to 9.07) |
| **Further procedures** | | | | | |
| Conservative surgery for endometriosis | 660.70 (1528.89) | 205 | 988.68 (1344.43) | 200 | 327.98 (27.98 to 585.83) |
| Hysterectomy | 420.66 (1173.08) | 205 | 597.24 (1314.11) | 200 | 176.58 (-47.59 to 437.96) |
| **Test/investigations** | | | | | |
| Laparoscopy | 226.28 (701.81) | 205 | 162.73 (550.69) | 200 | -63.56 (-195.26 to 63.58) |
| Ultrasound scan | 3.07 (10.91) | 205 | 5.15 (17.08) | 200 | 2.08 (-0.28 to 5.25) |
| Hysteroscopy | 9.01 (73.05) | 205 | 0 (0) | 200 | -9.01 (-22.80 to -2.74) |
| **Productivity loss** | | | | | |
| Days taken off-paid work | 2,608.16 (1388.07) | 205 | 2,848.04 (1225.99) | 200 | -239.88  (-468.36 to 10.78) |

**Table S5. Mean total costs (£) (2021– 22 prices)**

| **Resource item** | **LAPs group (N=205)** | **COCP group (N=200)** | **Bootstrap difference,**  **mean cost difference (95% CI)** |
| --- | --- | --- | --- |
|  | **Mean (SD)** | **Mean (SD)** |  |
| Cost of healthcare visit | 458.49 (278.35) | 591.35 (513.71) | 132.86 (53.55 to 213.16) |
| Cost of medications | 159.20 (139.52) | 125.49 (153.59) | -33.70 (-60.96 to -0.27) |
| Cost of further procedures | 1081.36 (2024.50) | 1585.92 (2111.36) | 504.56 (108.25 to 931.66) |
| Cost of test/investigations | 238.36 (707.14) | 167.87 (550.19) | -70.49 (-190.48 to 47.91) |
| Total costs of health service use | 1937.41 (2375.22) | 2470.64 (2358.75) | 533.23 (42.17 to 1008.77) |

**Table S6. Additional deterministic sensitivity analysis**

|  | **Mean cost** | **Mean effect (QALY)** | **Bootstrap difference, mean incremental cost (95% CI)** | **Bootstrap difference, mean incremental effect (95% CI)** | **ICER** |
| --- | --- | --- | --- | --- | --- |
| **Base case analysis** | | | | | |
| LAPs | £1,937 | 1.936 | £533 (£52 to £984) | 0.031 (-0.079 to 0.139) | £17,193 |
| COCP | £2,470 | 1.968 |  |  |  |
| **Complete case analysis** | | | | | |
| LAPs | £1,937 | 1.970 | £535 (£-301 to £1,317) | -0.021 (-0.137 to 0.134) | Dominated |
| COCP | £2,472 | 1.949 |  |  |  |

**Table S7. The secondary outcomes response rates**

| **Time point** | **LAPs group (N=205),**  **n (%)** | **COCP group (N=200),**  **n (%)** | **Total** | **Missing** |
| --- | --- | --- | --- | --- |
| **ICECAP-A questionnaire** | | | | |
| Baseline | 195 (95.12) | 192 (96) | 387 | 18 |
| Month 6 | 162 (79.02) | 151 (75.5) | 313 | 92 |
| Month 12 | 152 (74.15) | 152 (76) | 304 | 101 |
| Month 24 | 124 (60.49) | 110 (55) | 234 | 171 |
| Month 36 | 100 (48.78) | 97 (48.5) | 197 | 208 |
| Complete case baseline to month 36 | 76 (37.07) | 71 (35.5) | 147 | 258 |
| **EHP-30 pain domain questionnaire** | | | | |
| Baseline | 197 (96.09) | 192 (96) | 389 | 16 |
| Month 6 | 162 (79.02) | 150 (75) | 312 | 93 |
| Month 12 | 150 (73.17) | 153 (76.5) | 303 | 102 |
| Month 24 | 157 (76.59) | 140 (70) | 297 | 108 |
| Month 36 | 173 (84.39) | 164 (82) | 337 | 68 |
| Complete case baseline to month 36 | 113 (55.12) | 99 (49.5) | 212 | 193 |

**Table S8. Secondary outcomes outcome results**

| Time point | LAPs group (N=205) | | COCP group (N=200) | | Bootstrap adjusted mean difference (95% CI) |
| --- | --- | --- | --- | --- | --- |
|  | n | Mean (SD) | n | Mean (SD |  |
| Years of full capability (YFC) estimates: ICECAP-A scores | | | | | |
| Baseline ICECAP-A score | 195 | 0.802 (0.172) | 192 | 0.800 (0.175) | -0.002  (-0.038 to 0.032) |
| Month 6-ICECAP-A score | 162 | 0.792 (0.181) | 151 | 0.802 (0.176) | 0.011  (-0.019 to 0.044) |
| Month 12-ICECAP-A score | 152 | 0.811 (0.179) | 152 | 0.791 (0.213) | -0.017  (-0.054 to 0.021) |
| Month 24-ICECAP-A score | 124 | 0.810 (0.193) | 110 | 0.816 (0.186) | 0.0101  (-0.033 to 0.051) |
| Month 36-ICECAP-A score | 100 | 0.830 (0.161) | 97 | 0.774 (0.225) | -0.0275  (-0.067 to 0.016) |
| Total complete case YFCs | 76 | 2.419 (0.353) | 71 | 2.309 (0.495) | -0.0254  (-0.116 to 0.0648) |
| Total imputed YFCs | 205 | 2.326 (0.389) | 200 | 2.320 (0.454) | -0.0034  (-0.0562 to 0.0525) |
| Pain score estimates: EHP-30 Pain Domain scores | | | | | |
| Baseline | 197 | 56.587 (17.330) | 192 | 55.753 (19.941) | -0.835  (-4.6601 to 2.671) |
| Month 6 | 162 | 35.045 (25.580) | 150 | 38 (26.376) | 2.165  (-2.804 to 7.129) |
| Month 12 | 150 | 35.061 (26.353) | 153 | 37.493 (25.358) | 2.697  (-2.166 to 7.811) |
| Month 24 | 157 | 32.121 (26.171) | 140 | 33.555 (26.487) | 1.477  (-4.034 to 6.932) |
| Month 36 | 173 | 32.948 (24.950) | 164 | 32.858 (27.552) | 0.018  (-4.794 to 5.023) |
| Complete case pain score reduction from baseline to month 36 | 113 | 26.106 (23.910) | 99 | 22.062 (24.381) | -0.969  (-6.276 to 3.635) |
| Imputed pain score reduction from baseline to month 36 | 205 | 23.549 (22.060) | 200 | 23.403 (23.673) | -0.145  (-4.509 to 4.182) |
