# Supplementary material for: Cost-effectiveness of long-acting progestogens versus the combined oral contraceptives pill for preventing recurrence of endometriosis-related pain following surgery: an economic evaluation alongside the PRE-EMPT trial
Source: BMJ Open. 2024 Dec 9;14(12):e088072. doi: 10.1136/bmjopen-2024-088072 (PMC11628948; doi:10.1136/bmjopen-2024-088072)
Supplement: Supplementary file 2 [file bmjopen-2024-088072.reviewer_comments.pdf]

PEER REVIEW HISTORY

BMJ Open publishes all reviews undertaken for accepted manuscripts. Reviewers are asked to complete a checklist review form and are provided with free text boxes to elaborate on their assessment. These free text comments are reproduced below.

ARTICLE DETAILS

Title (Provisional)

The cost-effectiveness of long-acting progestogens versus the combined oral contraceptive pill for preventing recurrence of endometriosis-related pain following surgery: An economic evaluation alongside the PRE-EMPT trial

Authors

Melyda, MELYDA; Monahan, Mark; Cooper, Kevin; Bhattacharya, Siladitya; Daniels, Jane; Cheed, Versha; Middleton, Lee; Roberts, Tracy

---

VERSION 1 - REVIEW

**Reviewer**	**1**
**Name**	**Pasvol, Thomas**
**Affiliation**	**University College London, The Research Department of Primary Care and Population Health**
**Date**	**17-May-2024**
**COI**	**None**

This is a generally well written manuscript which adds to our understanding on the topic. My major concern is the decision to group LNG-IUD and DMPA together despite the obvious differences in how these treatments work and strikingly different cost. This is likely to have diluted out some meaningful findings. Although contraception and women's health are my specialist areas, I have limited experience with cost-effectiveness analyses. I would advise that the journal seeks an additional opinion to ensure that the statistical methodology used is appropriate.

MAJOR:

1)

Mean cost differs enormously between the LNGIUD and DMPA groups and they are completely different ways to deliver progestogens. It seems unusual to group them together for the primary analysis. From your data, perhaps DMPA is more cost effective than either COCP or LNG-IUD. It will clearly be a very large undertaking to redo the study with three

groups but I do feel that subgroup analysis five should be explored in far greater depth prior to publication.

2)

Throughout the manuscript (including the abstract), many of the 95% CIs cross the null value but the authors have interpreted the results as favouring one treatment over the other (e.g. P4 line 16, p14 line 1-7). The authors should acknowledge that their results may indicate no difference between groups at the 95% threshold, or that their study is underpowered.

3)

I can't work out if you have captured all healthcare utility required for contraceptive provision? Not all women get their contraception from the GP. Did you capture visits to family planning clinics, GUM clinics and integrated sexual health centres for repeat CHC scripts or DMPA injections? If not, suggest including as a limitation.

MINOR:

4)

Authors should be consistent with use of numbers <10 in sentences throughout the manuscript, some are given as numerals '5' and others as words 'three'.

5)

P5 line 16

In the UK, the new preferred term for a LNGIUS is levonorgestrel intrauterine device (LNG-IUD). 'intrauterine system' is being phased out. See: FSRH Clinical Guideline: Intrauterine contraception (March 2023, Amended July 2023) - Faculty of Sexual and Reproductive Healthcare Please update throughout the manuscript.

6)

P5 line 13-17

This paragraph contains grammatical errors 'which requiring daily oral intake', 'which administered every three months'.

7)

P5 Line 17. Mirena is now licenced up to eight years for contraception. Although, the licence remains five years for heavy menstrual bleeding, it could provide symptomatic benefits in some patients for longer. Please rephrase.

**Reviewer**	**2**
**Name**	**Png, May Ee**

Affiliation **University of Oxford, Nuffield Department of Primary Care Health Sciences**

Date **13-Aug-2024**

COI **None**

This is an economic evaluation comparing the cost-effectiveness of LAPs (LINGIUS or DMPA) and COCP among women who underwent conservative endometriosis surgery under the NHS perspective over a 36-month time horizon. Outcomes investigated were QALYs, years of full capability, pain score reduction and treatment failure avoided. However, I have the following concerns.

Methods: It would still be good to briefly describe the population as recommended by the CHEERS 2022 reporting guideline. Were there no exclusion criteria?

Methods: What is the reason for not using the latest crosswalk value set recommended by NICE? NICE clearly stated "The mapping function developed by the Decision Support Unit (Hernández Alava et al. 2017), using the 'EEPRU dataset' (Hernández Alava et al. 2020), should be used for reference-case analyses.".

Methods: How was the EHP-30 scored?

Methods: Was a HEAP written before the analysis? If not, please state so in the paper as recommended by the CHEERS 2022 reporting guideline.

Methods: How can item #12 of the CHEERS 2013 checklist be "N/A" when QALYs was used in this study?

Methods: Please follow the instruction from BMJ Open and say that CHEERS reporting guideline was used. It would be good to use the 2022 checklist instead of the 2013 checklist that was submitted.

Discussion: "All the sensitivity and/or subgroup analyses support the result" But complete case analysis showed that CCOP was dominated when compared to LAPs.

Discussion: Perhaps the authors can discuss the limitation of not adopting the NICE recommended NHS and personal social services perspective?

Implication for policy: If COCP has been shown to not be cost-effective relative to LAPs when natural units were considered as the outcome, is it still accurate to conclude that the "trial-based economic evaluation suggests a higher probability of COCP being cost-effective compared to the LAPs"?

VERSION 1 - AUTHOR RESPONSE

Reviewers' comments

Reviewer 1. Dr. Thomas Pasvol, University College London

This is a generally well written manuscript which adds to our understanding on the topic. My major concern is the decision to group LNG-IUD and DMPA together despite the obvious differences in how these treatments work and strikingly different cost. This is likely to have diluted out some meaningful findings. Although contraception and women's health are my specialist areas, I have limited experience with cost-effectiveness analyses. I would advise that the journal seeks an additional opinion to ensure that the statistical methodology used is appropriate.

MAJOR:

1. Mean cost differs enormously between the LNG-IUS and DMPA groups and they are completely different ways to deliver progestogens. It seems unusual to group them together for the primary analysis. From your data, perhaps DMPA is more cost effective than either COCP or LNG-IUS. It will clearly be a very large undertaking to redo the study with three groups, but I do feel that subgroup analysis five should be explored in far greater depth prior to publication.

Response: Thank you for your feedback. We acknowledge that LNG-IUS and DMPA have different mechanisms of action and costs. However, our approach was guided by the design of the PRE-EMPT trial, which evaluated LAPs as a class of treatments compared to the COCP.[1]

The rationale for this grouping was based on several factors:

- Trial design: The PRE-EMPT trial was designed as a pragmatic, parallel group study comparing LAPs as a class to COCP. As this study is a trial-based economic evaluation, we were constrained by the design and objectives of the trial, which guided our approach.
- Sample size and statistical power: Analysing LNG-IUS and DMPA separately would have reduced the sample size for each subgroup, potentially limiting the statistical power to detect meaningful differences in cost-effectiveness.
- Clinical effectiveness: As reported in our published clinical trial paper, the primary analysis demonstrated similar effectiveness between LAPs and COCP in preventing recurrence of endometriosis-related pain.[1] This supports the validity of considering LAPs as a group for the economic evaluation.

To address this, we have included subgroup analyses in our study (page 9, lines 13-14), which provide insights into potential differences between LNG-IUS and DMPA versus COCP while maintaining the overall focus on LAPs as a class of treatments. However, conducting an in-depth comparison between different types of LAPs was beyond the scope of our current study and the design of the PRE-EMPT trial. We appreciate the reviewer's comment, which has helped clarify the focus and limitations of our study. We have now added the text below as a limitation in our discussion.

Finally, a detailed LAP subgroup analysis comparing LNG-IUS and DMPA to determine the more cost-effective option was not considered in this study, as it was beyond the scope of our primary research question and the design of the PRE-EMPT trial. (Page 15, lines 8-11)

---

[1] Cooper K G, Bhattacharya S, Daniels J P, Horne A W, Clark T J, Saridogan E et al. Long acting progestogens versus combined oral contraceptive pill for preventing recurrence of endometriosis related pain: the PRE-EMPT pragmatic, parallel group, open label, randomised controlled trial *BMJ* 2024; 385 :e079006 doi:10.1136/bmj-2023-079006.

2. Throughout the manuscript (including the abstract), many of the 95% CIs cross the null value but the authors have interpreted the results as favouring one treatment over the other (e.g. P4 line 16, p14 line 1-7). The authors should acknowledge that their results may indicate no difference between groups at the 95% threshold, or that their study is underpowered.

   Response: Thank you for this comment and suggestion. The interpretation of 95% confidence intervals in economic evaluations/health economics studies differs from traditional hypothesis testing in clinical studies. Health economics analysis is required to follow CHEERS reporting guidelines[2]. These guidelines require that uncertainty around the mean is estimated through sampling and simulation. This contrasts with clinical guidelines, which focus on p-values and statistically significant differences.

   In economic evaluations, the focus is on decision-making rather than hypothesis testing. The goal is to provide an estimate of cost-effectiveness that is most informative for decision-making, rather than determine statistical significance. Many studies in the literature have also addressed this issue.[3,4,5,6] This is why we have interpreted the results as favouring one treatment over the other in certain instances. The presentation of results as presented by our probabilistic sensitivity analysis and depicted by the cost effectiveness plane (Figure 1) and the cost effectiveness acceptability curve (Figure 2) is accepted by NICE[7] and acknowledged in its decision-making.

3. I can't work out if you have captured all healthcare utility required for contraceptive provision? Not all women get their contraception from the GP. Did you capture visits to family planning clinics, GUM clinics and integrated sexual health centres for repeat CHC scripts or DMPA injections? If not, suggest including as a limitation.

   Response: Thank you for the questions and suggestion. Since this is a trial-based study, it was designed to capture all relevant outcomes and healthcare utilisation. The trial was structured to monitor all aspects of care the women received within the trial, including contraception/treatment they accessed. While, in real life, women may obtain contraception from various sources or settings, initial treatment was administered or fitted at the hospital or provided through prescription advice to GPs. Future treatments—whether changes to or continuation of depo, COCP, or removal or fitting of LNG-IUS—were at the patient's discretion. Our only way of determining contraceptive treatment at the three-year follow-up was through patient self-reporting via a resource use questionnaire. We recognise that this data may be unknown for up to 20% of participants, but randomisation should balance this across groups.

---

[2] Husereau D, Drummond M, Augustovski F, et al. Consolidated Health Economic Evaluation Reporting Standards 2022 (CHEERS 2022) Explanation and Elaboration: A Report of the ISPOR CHEERS II Good Practices Task Force. Value Health 2022;25. doi:10.1016/j.jval.2021.10.008
[3] O'Brien BJ, Briggs AH. Analysis of uncertainty in health care cost-effectiveness studies: an introduction to statistical issues and methods. Statistical methods in medical research. 2002 Dec;11(6):455-68.
[4] Whitehurst DG, Bryan S. Trial-based clinical and economic analyses: the unhelpful quest for conformity. Trials 2013;14:421.
[5] Betensky, R. A., & Newberger, N. G (2019) New Guidelines for Statistical Reporting. *N Engl J Med* **381**:16, 1597-1598.
[6] Claxton K. The irrelevance of inference: a decision-making approach to the stochastic evaluation of health care technologies. J Health Econ. 1999 Jun;18(3):341-64. doi: 10.1016/s0167-6296(98)00039-3. PMID: 10537899.
[7] NICE. NICE Health Technology Evaluations: The Manual. https://www.nice.org.uk/process/pmg36/resources/nice-health-technology-evaluations-the-manual-pdf-72286779244741 (2022)

As the resource use questionnaire used to capture any healthcare resources outside the trial setting relies on self-reporting we acknowledge the potential for under-reporting, which we have noted in our limitations (page 14, lines 36-37).

MINOR:

4. Authors should be consistent with use of numbers <10 in sentences throughout the manuscript, some are given as numerals '5' and others as words 'three'.

Response: Thank you for pointing this out. We have revised the manuscript to ensure consistent use of numerals and words for numbers below 10 throughout.

Surgical treatment is the predominant treatment for alleviating endometriosis-related pain and symptoms but it is associated with a high recurrence rate leading to repeat surgery for 27% of patients within *five* year. (Page 4, line 12)

All the outcome measures were collected at baseline, *six*, 12, 24, and 36 months after randomisation. (Page 5, line 7)

Health-care resource utilization data were gathered alongside the trial at various intervals: baseline, *six*, 12-, 24-, and 36-months post-randomisation… (Page 5, line 41)

In terms of limitations, it is possible that the *three* year follow-up duration may not fully capture the chronic nature of endometriosis,… (Page 14, line 34)

5. P5 line 16: In the UK, the new preferred term for a LNGIUS is levonorgestrel intrauterine device (LNG-IUD). 'intrauterine system' is being phased out. See: FSRH Clinical Guideline: Intrauterine contraception (March 2023, Amended July 2023) - Faculty of Sexual and Reproductive Healthcare Please update throughout the manuscript.

   Response: Thank you for bringing this to our attention. We acknowledge the recent shift in preferred terminology to levonorgestrel intrauterine device (LNG-IUD), as outlined in the FSRH Clinical Guideline: Intrauterine contraception (March 2023, amended July 2023). However, when the trial was completed, the term 'IUS' was still in use, and this term is also used consistently across all published protocols, clinical papers, and the HTA monograph. Therefore, we believe it is important to maintain consistency throughout the manuscript.

6. P5 line 13-17: This paragraph contains grammatical errors 'which requiring daily oral intake', 'which administered every three months'.

Response: Thank you for pointing out these grammatical errors. We have corrected this sentence.

This includes the combined oral contraceptive pill (COCP) which *requires* daily oral intake, and long-acting progestogens (LAPs), such as depot medroxyprogesterone acetate (DMPA) which *is* administered every three months, and the levonorgestrel-releasing intrauterine system (LNG-IUS). (Page 4, lines 13-16)

7. P5 Line 17: Mirena is now licenced up to eight years for contraception. Although, the licence remains five years for heavy menstrual bleeding, it could provide symptomatic benefits in some patients for longer. Please rephrase.

   Response: Thank you for this update. We have revised the manuscript to indicate that LNG-IUS is now licensed for up to eight years for contraception and up to five years for heavy menstrual bleeding (see below). However, since there are no extension studies beyond five years to conclude that it still provides benefits (e.g. endometrial gland suppression) after this period, we believe it is best not to discuss this in the current study.

   This includes the combined oral contraceptive pill (COCP) which requires daily oral intake, and long-acting progestogens (LAPs), such as depot medroxyprogesterone acetate (DMPA) which is administered every three months, and the levonorgestrel-releasing intrauterine system (LNG-IUS). *The LNG-IUS is licensed for up to eight years for contraception and up to five years for heavy menstrual bleeding.*(Page 4, Lines 16-18)

Reviewer 2. Dr. May Ee Png, University of Oxford

This is an economic evaluation comparing the cost-effectiveness of LAPs (LINGIUS or DMPA) and COCP among women who underwent conservative endometriosis surgery under the NHS perspective over a 36-month time horizon. Outcomes investigated were QALYs, years of full capability, pain score reduction and treatment failure avoided. However, I have the following concerns.

1. Methods: It would still be good to briefly describe the population as recommended by the CHEERS 2022 reporting guideline. Were there no exclusion criteria?

   Response: Thank you for this feedback. We have included a brief description of the participant population and exclusion criteria in the revised manuscript, as seen in text below. For a more comprehensive account of the trial details and participant selection criteria, we have cited our published clinical paper (page 4, line 39).

   The PRE-EMPT trial was a multicentre, pragmatic, parallel-group, open-label, randomised controlled trial. Details of the trial *including the economic evaluation plan and protocol* have been published elsewhere.[11-13] Briefly, 405 women *aged 16-45 years with symptoms suggestive of endometriosis and scheduled for diagnostic laparoscopy with concurrent or previous conservative surgery* were recruited and randomised across 34 hospitals in the UK from November 2015 to March 2019, of which 205 receiving LAPs (91 to LNG-IUD and 114 to DMPA) and 200 receiving COCP. *Exclusion criteria included infertility, immediate plans to conceive, elective surgery for deep disease or endometrioma, contraindications to hormonal treatment, and suspicion of malignancy.*[11,13] (page 4, line 31-45)

2. Methods: What is the reason for not using the latest crosswalk value set recommended by NICE? NICE clearly stated "The mapping function developed by the Decision Support Unit (Hernández Alava et al. 2017), using the 'EEPRU dataset' (Hernández Alava et al. 2020), should be used for reference-case analyses.".

   Response: Thank you for your detailed feedback. We initially followed the protocol outlined in our published statistical analysis plan. However, we recognise the importance of adhering to NICE recommendations for mapping EQ-5D-5L to EQ-5D-3L using the latest crosswalk value set. Consequently, we have re-conducted the analysis using the recommended crosswalk value set.

   Although we did not find substantial differences in the results, we have revised the results throughout the manuscript to reflect this updated approach. The main findings remain consistent: COCP is more expensive but leads to a higher QALYs gained compared to LAPs.

   Revisions have been made in below section:

   We have updated Table 2 and Figure 1,2 (Page 11)

   Abstract… Results
   For the primary analysis, the COCP group incurred an additional cost of £533 (95% CI £52 to £983) per woman compared to LAPs. Treatment with COCP generated additional QALYs of 0.0*31* (95% CI -0.0*79* to 0.1*39*) compared to the LAPs group, over 36 months follow-up. The

incremental cost effectiveness ratio for COCP compared to LAPs is therefore approximately *£17,193* per QALY. The probabilistic sensitivity analysis suggested there was a *54.7%* probability that COCP would be cost-effective at the £20,000/QALY threshold, indicating considerable uncertainty regarding which treatment is more cost-effective. The secondary analyses revealed results more in favour of LAPs. (Page 2, line 22-28)

At baseline, participants in the LAPs group had a slightly lower average starting EQ-5D-5L score than those in COCP group (0.6*38* and 0.6*45*, respectively). However, by 36 months, participants in the LAPs group showed a slightly higher score compared to those in the COCP group (0.69*3* and 0.68*4*, respectively). The mean adjusted imputed QALY difference between the two groups was 0.0*31* (95% CI -0.0*79* to 0.1*39*), favouring the COCP group. (Page 10, lines 1-5)

… Thus, the COCP group was estimated to be £533 (95% CI 52 to 983) per woman more costly but offered slightly higher QALYs by 0.0*31* (95% CI -0.0*79* to 0.1*39*) compared to the LAPs group, over the 36 months follow-up. This resulted in an ICER of *£17,193* per QALY. (Page 10, line 16-19)

… The cost effectiveness acceptability curve (CEAC) (Figure 1b) shows the probability for the COCP intervention being considered cost-effective is approximately *54.7%* at threshold of £20,000 per QALY. (Page 10, line 30)

….. The difference (£533, 95% CI £52 to £983) is primarily due to the cost of further surgeries for endometriosis or hysterectomy. COCP yields a slight increase in QALYs of 0.0*31* (95% CI -0.0*79* to 0.1*39*) over 36 months. Thus, the incremental cost-effectiveness ratio for treatment with COCP compared to LAPS is £*17,193* per QALY……. *However, the probabilistic sensitivity analysis indicated a 54.7% probability that COCP is more cost effective than LAPs in terms of cost per QALY gained. This probability is only slightly above 50%, highlighting a considerable level of uncertainty regarding which treatment is truly more cost-effective.* (Page 14, lines 5-13)

3. Methods: How was the EHP-30 scored?

Response: Thank you for this question. The EHP-30 pain domain was scored from 0 (best outcome) to 100 (worst outcome), based on 11 questions. The change in score was calculated as the difference between baseline and the 36-month follow-up. We have added more detail in the manuscript.

The secondary analysis also considered the differences in pain scores, which was determined by the changes in the EHP30 pain domain score from the baseline to 3 years follow-up. The EHP-30 questionnaire is a patient-reported outcome measure to assess health-related quality of life in endometriosis.[18] The core components of this instrument encompass pain, control and powerlessness, social support, emotional well-being, and self-image scale scores. *Only the pain-domain score was considered for the secondary analysis outcome. The pain domain consists of 11 questions, with overall zero as the best outcome to 100 pain score as worst score.[19] The pain score changing was the difference between the pain score at the baseline and at the 36 months follow up time.* (page 5, lines 31-34)

4. Methods: Was a HEAP written before the analysis? If not, please state so in the paper as recommended by the CHEERS 2022 reporting guideline.

   Response: Thank you for your query. While a detailed HEAP was not explicitly written, we followed the published economic evaluation protocol for the PRE-EMPT trial, which some relevant aspects of the HE analysis plan and we have referenced it in the manuscript (Page 4, line 38). We have added this into the CHEERS 2022 checklist.

   In the protocol, we initially planned to conduct a model-based economic evaluation. However, the decision not to proceed with this approach was deliberate and primarily influenced by the nature of the trial. While an attempt was made, it proved to be infeasible due to the excessive assumptions required. Since we had access to trial data, the trial team agreed that a trial-based analysis would provide the most robust assessment. The extended modelling approach presented unique challenges, particularly with crossover treatments, which introduced complexity and uncertainty—such as individual patient responses and physician recommendations—that were difficult to model accurately. As a result, the trial-based evaluation, relying on actual patient data and observed treatment patterns, was deemed a more reliable and relevant assessment of cost-effectiveness, avoiding potential pitfalls associated with complex assumptions in a model-based analysis.

5. Methods: How can item #12 of the CHEERS 2013 checklist be "N/A" when QALYs was used in this study?

   Response: Thank you for these comments. This was indeed an oversight. I mistakenly submitted the wrong version of the CHEERS checklist, although we had used and submitted the 2022 version for the NIHR HTA report[8] for this trial. We have now submitted the correct version with the revised manuscript. Thank you once again for pointing this out.

6. Methods: Please follow the instruction from BMJ Open and say that CHEERS reporting guideline was used. It would be good to use the 2022 checklist instead of the 2013 checklist that was submitted.

   Response: Thank you for the input. We have now submitted the correct version with the revised manuscript.

7. Discussion: "All the sensitivity and/or subgroup analyses support the result" But complete case analysis showed that CCOP was dominated when compared to LAPs.

   Response: Thank you for this feedback. Given that the complete case analysis included only 212 participants (52% of the total sample), we have decided to exclude this from the paper to prevent any potential confusion in interpreting the results.

---

[8] Cooper KG, Bhattacharya S, Daniels JP, Cheed V, Gennard L, Leighton L, et al. Preventing recurrence of endometriosis-related pain by means of long-acting progestogen therapy: the PRE-EMPT RCT. Health Technol Assess 2024;28(55)

8. Discussion: Perhaps the authors can discuss the limitation of not adopting the NICE recommended NHS and personal social services perspective?

Response: We apologies for the omission. The primary base-case economic analysis did adopt the NICE-recommended NHS and personal social services perspective. We have revised the main text to clarify this:

The primary base-case economic analysis adopted a cost-utility analysis (CUA) framework, conducted from the perspective of the UK National Health Service (NHS) *and personal social services.* (Page 8, lines 20-22)

9. Implication for policy: If COCP has been shown to not be cost-effective relative to LAPs when natural units were considered as the outcome, is it still accurate to conclude that the "trial-based economic evaluation suggests a higher probability of COCP being cost-effective compared to the LAPs"?

Response: Thank you for raising this point. We still found that the COCP has a slightly higher probability of being cost-effective compared to LAPs, but there remains considerable uncertainty regarding which treatment is more cost-effective. We have revised the sentence in the manuscript (as shown below) for clarity, ensuring it accurately reflects that the probability mentioned is based on the primary analysis and that cost-effectiveness is assessed in terms of cost per QALY gained.

While the primary trial-based economic evaluation suggests *slightly* higher probability of COCP being cost-effective compared to the LAPs *in terms of cost per QALY gained*, *there remains considerable uncertainty regarding which treatment is more cost-effective*. *Additionally*, *COCP group is also* associated with an increased risk of further *major* surgery *for recurrent endometriosis and hysterectomy,* which *may influence decision making of* women and their health care practitioners. (Page 15, lines 28-33)

VERSION 2 - REVIEW

**Reviewer**	**1**
**Name**	**Pasvol, Thomas**
**Affiliation**	**University College London, The Research Department of Primary Care and Population Health**
**Date**	**01-Nov-2024**
**COI**	

Thank you for addressing my comments completely.

**Reviewer**	**2**
**Name**	**Png, May Ee**
**Affiliation**	**University of Oxford, Nuffield Department of Primary Care Health Sciences**
**Date**	**21-Oct-2024**
**COI**	

The authors have adequately addressed my concerns except the following:

1. An economic evaluation section within the study protocol is different to a HEAP because it lacks details and as a good practice, follow this guideline by Thorn et al (https://www.sciencedirect.com/science/article/pii/S1098301520344442). Please correct the CHEERS checklist to indicate that no HEAP was written.

2. The authors removed the complete case analysis because "complete case analysis included only 212 participants (52% of the total sample)" but if a complete case analysis was defined in the protocol for the economic evaluation, I do not support removing complete case analysis especially when it reported different results from the other analyses because it falls under "questionable research practice" and there is bias from selective reporting.

And even if a complete case analysis was not explicitly written in the protocol, as a minimum, it should be written in the Discussion section that the final analysis is different from the planned analysis and the reason for the difference (i.e. removing complete case analysis).

VERSION 2 - AUTHOR RESPONSE

Reviewers' comments

Reviewer 1. Dr. Thomas Pasvol, University College London

Thank you for addressing my comments completely.

Response: Thank you for your positive feedback. We are glad that the revisions met your expectations.

Reviewer 2. Dr. May Ee Png, University of Oxford

The authors have adequately addressed my concerns except the following:
1. An economic evaluation section within the study protocol is different to a HEAP because it lacks details and as a good practice, follow this guideline by Thorn et al

(https://www.sciencedirect.com/science/article/pii/S1098301520344442). Please correct the CHEERS checklist to indicate that no HEAP was written.

Response: Thank you for your feedback. We have revised the CHEERS checklist to indicate that no HEAP was written.

2. The authors removed the complete case analysis because "complete case analysis included only 212 participants (52% of the total sample)" but if a complete case analysis was defined in the protocol for the economic evaluation, I do not support removing complete case analysis especially when it reported different results from the other analyses because it falls under "questionable research practice" and there is bias from selective reporting.
And even if a complete case analysis was not explicitly written in the protocol, as a minimum, it should be written in the Discussion section that the final analysis is different from the planned analysis and the reason for the difference (i.e. removing complete case analysis).

Response: Thank you for your concern regarding the complete case analysis. There was no complete case analysis specified in the protocol. In this case, we believe this analysis could lead to confusion rather than clarity for the reader, as it is based on only 52% of the sample. However, in response to your feedback, we have included the complete case analysis in the manuscript (see below) and presented the results in the supplementary materials (Table S6), with a summary in the results section. We opted not to provide additional context in the discussion, as we feel it may create further confusion and potentially mislead interpretations of the results.

Method section:
Deterministic sensitivity analysis
A series of deterministic sensitivity analyses were carried out to assess the robustness of the base-case results:

1. Undiscounted analysis – this analysis presented the undiscounted costs and outcomes
2. Partial societal perspective analysis – this analysis assessed the impact of including work-related costs of patients.
3. Additional analysis incorporating costs of other types of surgery mentioned by participants (removal of fibroids, removal of polyps, and endometrial ablation)
4. Subgroup analysis – this analysis evaluated the cost-effectiveness of the COCP with each of the LAP's subgroups: COCP versus LNG-IUS, and COCP versus DMPA
5. *Complete-case analysis – the analysis was re-run using only participants with complete cost and outcome data* (Page 9, lines 15-16)

Result section:
Table 2 presents the results of the deterministic sensitivity analysis. Across *most* analyses - COCP consistently yielded higher costs but greater QALYs compared to LAPs. These findings were also consistent with the subgroup analyses comparing COCP with LAPs subgroups. *However, in the complete case analysis (see Table S6), COCP was associated with higher costs and fewer QALYs, but this was limited by a smaller sample size and missing data, thereby affecting the robustness of this finding.* (Page 10, lines 26-31)

VERSION 3 - REVIEW

**Reviewer**	**2**
**Name**	**Png, May Ee**
**Affiliation**	**University of Oxford, Nuffield Department of Primary Care Health Sciences**
**Date**	**11-Nov-2024**
**COI**	

The authors have adequately addressed my concerns.
